# Nonlinear optical properties of photosensory core modules of monomeric and dimeric bacterial phytochromes

**DOI:** 10.1002/pro.70118

**Published:** 2025-04-18

**Authors:** Diana Galiakhmetova, Aleksandr Koviarov, Viktor Dremin, Tatjana Gric, Dmitrii Stoliarov, Andrei Gorodetsky, Marios Maimaris, Daria M. Shcherbakova, Mikhail Baloban, Vladislav V. Verkhusha, Sergei G. Sokolovski, Edik Rafailov

**Affiliations:** ^1^ Aston Institute of Photonic Technologies Aston University Birmingham UK; ^2^ Department of Electronic Systems Vilnius Gediminas Technical University Vilnius Lithuania; ^3^ School of Physics and Astronomy University of Birmingham Birmingham UK; ^4^ Ultrafast Optoelectronics Group Imperial College London London UK; ^5^ Department of Genetics and Gruss‐Lipper Biophotonics Center Albert Einstein College of Medicine Bronx New York USA; ^6^ Medicum, Faculty of Medicine University of Helsinki Helsinki Finland

**Keywords:** bacterial phytochrome, DrBphP, Near‐infrared fluorescent protein (iRFP), long‐wavelength excitation, two‐photon photoconversion

## Abstract

Near‐infrared (NIR) fluorescent proteins and optogenetic tools derived from bacterial phytochromes' photosensory core modules (PCMs) operate within the first (NIR‐I) tissue transparency window under single‐photon activation. Leveraging two‐photon (2P) light in the second transparency window (NIR‐II) for photoswitching bacterial phytochromes between Pr and Pfr absorption states offers significant advantages, including enhanced tissue penetration, spatial resolution, and signal‐to‐noise ratio. However, 2P photoconversion of bacterial phytochromes remains understudied. Here, we study the non‐linear Pr to Pfr photoconversion's dependence on irradiation wavelength (1180–1360 nm) and energy fluence (41–339 mJ/cm^2^) for the PCM of *Dr*BphP bacterial phytochrome. Our findings reveal substantially higher photoconversion efficiency for the engineered monomeric *Dr*BphP‐PCM (73%) compared to the natural dimeric *Dr*BphP‐PCM (57%). Molecular mechanical calculations, based on experimentally determined 2P absorption cross‐section coefficients for the monomer (167 GM) and dimer (170 GM), further verify these results. We demonstrate both short‐ (SWE) and long‐wavelength excitation (LWE) fluorescence of the Soret band using 405 and 810–890 nm laser sources, respectively. Under LWE, fluorescence emission (724 nm) exhibits saturation at a peak power density of 1.5 GW/cm^2^. For SWE, we observe linear degradation of fluorescence for both *Dr*BphP‐PCMs, decreasing by 32% as the temperature rises from 19 to 38°C. Conversely, under LWE, the monomeric *Dr*BphP‐PCM's brightness increases up to 182% (at 37°C), surpassing the dimeric form's fluorescence rise by 39%. These findings establish the monomeric *Dr*BphP‐PCM as a promising template for developing NIR imaging and optogenetic probes operating under the determined optimal parameters for its 2P photoconversion and LWE fluorescence.

## 1 INTRODUCTION

Modern fluorescence microscopy and optogenetics are powerful technologies for imaging and controlling biological processes with light at the cellular and molecular levels. Despite their immense potential in basic biology, translational studies, and drug discovery (Bhattacharya et al., 2014; Marcu et al., 2018; Mehta & Zhang, 2011; Wang et al., 2016; Zhang & Cohen, 2017), there are significant challenges and limitations associated with the non‐invasive use of these approaches *in vivo*. One of the primary challenges is the limited visible light penetration into biological tissues. Since the excitation light of commonly used fluorescent proteins and optogenetic tools is in the visible range, it exhibits large absorbance and scattering by tissues (Galiakhmetova et al., 2023). The high absorption by oxy‐ and deoxy‐hemoglobin limits the imaging of deeper tissue layers and anatomical structures, as it often leads to interference from overlying blood vessels. While melanin absorbs visible light that compromises image resolution and spatial precision of light activation in through‐skin studies, collagen, fat, and nervous tissues cause significant scattering of visible light as it traverses through them. For example, the brain is characterized by a double higher diffuse reflectance in the visible range compared to the longer wavelengths. These absorption and scattering effects contribute to image blurring, reduce spatial resolution, and limit light penetration depth.

**FIGURE 1:**
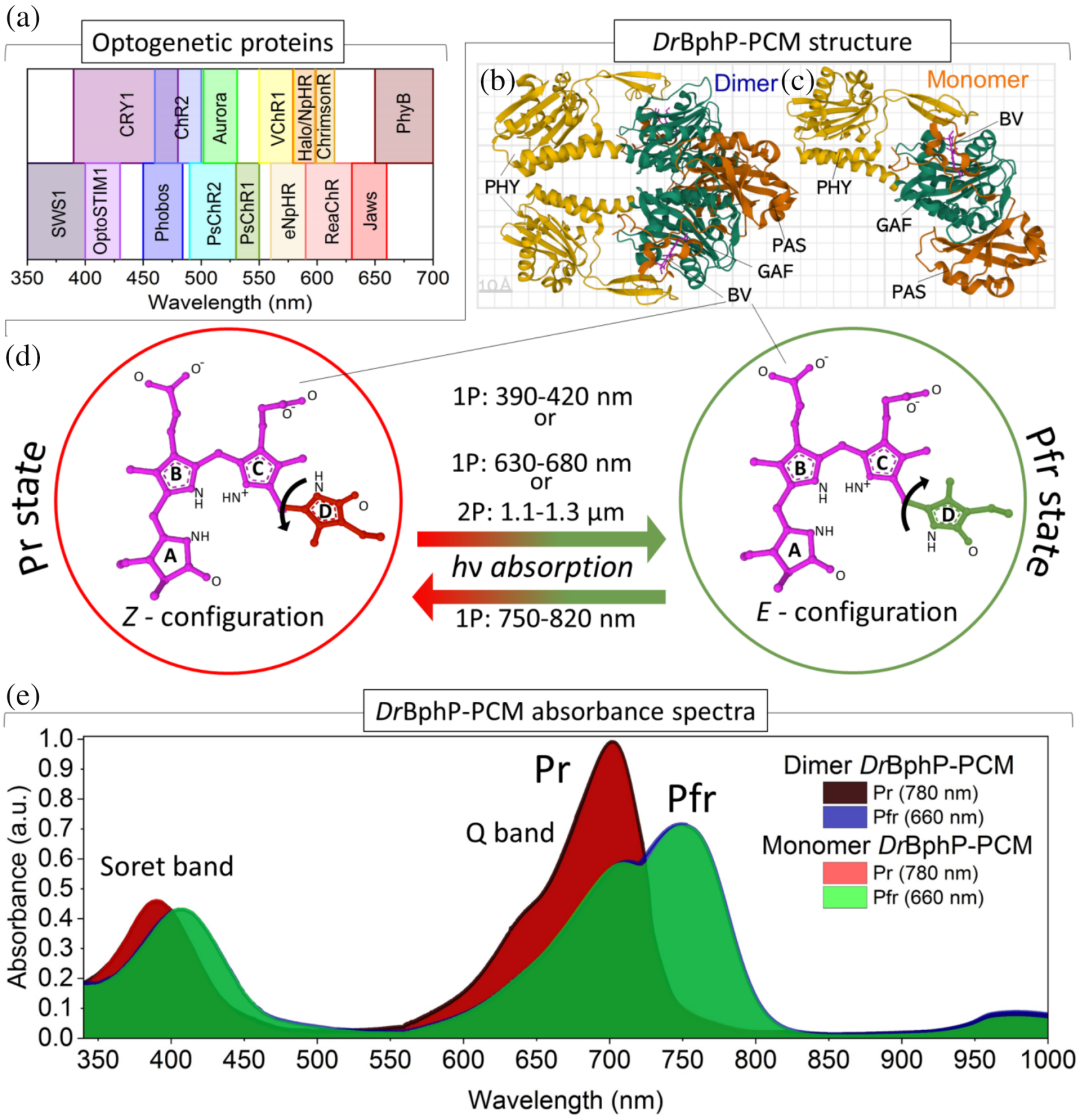
Light‐activatable proteins. (a) Excitation wavelength of various optogenetic proteins. (b) Structure of dimer and (c) monomer *Dr*BphP‐PCM with highlighted PAS, GAF, and PHY domains (Protein Data Bank (PDB) ID: 4Q0J (Burgie et al., 2014; Burgie et al., 2016)). (d) Schematic representation of *Z*‐ and *E*‐configurations of biliverdin IXα with photoconversion conditions. (e) Single‐photon (1P) absorption spectra of Pr (brown, red hues) and Pfr (blue, green hues) states of dimeric and monomeric photosensory core modules (PCMs) (concentration of 60 μм).

In optogenetics, substantial progress has recently been made in addressing the limitations of *in vivo* non‐invasive optical manipulation. The discovery and genetic engineering of a diverse array of light‐sensitive channelrhodopsin proteins, such as ReaChR, Jaws, and ChRmine (Figure 1a), resulted in customizing them to increase the duration of their active states or to respond to longer wavelengths (570–670 nm) (Chuong et al., 2014; Gradinaru et al., 2008; Grümbel et al., 2024; Kishi et al., 2022; Lin et al., 2013). Additionally, phytochrome photoreceptors of various origins, including algae, bacteria, and plants, have become molecular templates to engineer fluorescent proteins, biosensors, and optogenetic tools, which operate in the first near‐infrared (NIR‐I) tissue transparency window (650–900 nm) with a low hemoglobin and water absorbance (Auldridge & Forest, 2011; Chernov et al., 2017; Fomicheva et al., 2019; Shcherbakova et al., 2018). They demonstrate NIR‐I fluorescence emission stability under up to 720 nm single‐photon (1P) light excitation, repeatedly transitioning between bright and negligible fluorescent states (Lychagov et al., 2016).

However, even deeper tissue penetration and lower autofluorescence are possible by moving further to a second near‐infrared (NIR‐II) biological window of 1000–1350 nm (Galiakhmetova et al., 2023). NIR‐II imaging attracts great attention to monitoring deeper biological and pathological processes with a high signal‐to‐noise ratio due to a low level of tissue scattering (Gschwend et al., 2020; Li et al., 2020). The shift to NIR‐II has become achievable due to discoveries of 2P photoconversion and fluorescence excitation of rhodopsin‐based optogenetic tools at 1000–1050 nm (Tong et al., 2020; Yang et al., 2018) and phytochromes at 880–1310 nm (Piatkevich et al., 2017; Sokolovski et al., 2021).

Phytochromes' properties, such as sensitivity to longer wavelengths, long‐wavelength fluorescence emission, prolonged time of their two photochromic states, and the ability for fast photoconversion between them, make them attractive molecular templates for developing imaging probes and optogenetic tools (Chernov et al., 2017; Fomicheva et al., 2019; Shcherbakova et al., 2018). Notably, a subclass of bacterial phytochromes exhibits the longest absorbance wavelengths due to their biliverdin IXα (BV) chromophore (Figure 1b–d), having the largest electron‐conjugated system (Gourinchas et al., 2019). BV is a linear tetrapyrrole compound, consisting of four pyrrole rings (A–D) connected by methine bridges (Figure 1d). It is an enzymatic product of heme catabolism. In contrast to the phytochromobilin and phycocyanobilin chromophores required for plant and cyanobacterial phytochrome types, BV is naturally produced in various mammalian cells. That is why fluorescent proteins engineered from bacterial phytochromes allow the visualization and tracking of tagged molecules in cells and labeled cells in tissues without a supply of exogenous BV (Chernov et al., 2017).

Bacterial phytochromes, such as *Dr*BphP from *Deinococcus radiodurans*, typically have a molecular weight of 85–120 kDa and form tight head‐to‐head dimers. However, their photosensory core modules (PCMs), consisting of PAS‐GAF‐PHY domains with preserved light‐absorbing and photoswitching properties, are notably smaller (55–60 kDa) (Shcherbakova et al., 2018). The dimeric *Dr*BphP‐PCM protein will be referred to below as a “dimer” Each protomer (monomer) in the dimer incorporates the BV molecule in a binding pocket in the GAF domain and binds it covalently via a thioether bond with the N‐terminal sequence (NTS) (Figure 1b). The interaction between two protomers mainly occurs via hydrophobic interfaces of the GAF and PHY domains. In the *Dr*BphP‐PCM, light causes conformational changes in the GAF and PHY domains but does not cause dimer dissociation.

For experimental study and comparison of optical parameters, we made a mutagenesis of the interface between two protomers, resulting in the functional monomer *Dr*BphP and, correspondingly, its PCM (Matlashov et al., 2020), which we will refer to below as a “monomer”. It has an identical structure to a dimer's protomer (Figure 1c). The twice smaller size of the PCM monomer compared to the dimer (45 Å by 93 Å) minimizes possible steric hindrance and interference with proteins of interest genetically tagged with PCMs.

Despite numerous studies of monomer phytochromes (Auldridge et al., 2012; Takala et al., 2015, 2016), their nonlinear optical properties governing 2P conformational changes as well as short‐ (SWE) and long‐wavelength excitation (LWE) fluorescence remain unclear. Here, we theoretically and experimentally study 1P and 2P photoconversion and fluorescence emission of the *Dr*BphP‐PCM monomer and dimer and discuss possible mechanisms behind their optical behavior.

## 2 RESULTS

### 2.1 Photoconversion

#### 2.1.1 Linear optical properties

**FIGURE 2:**
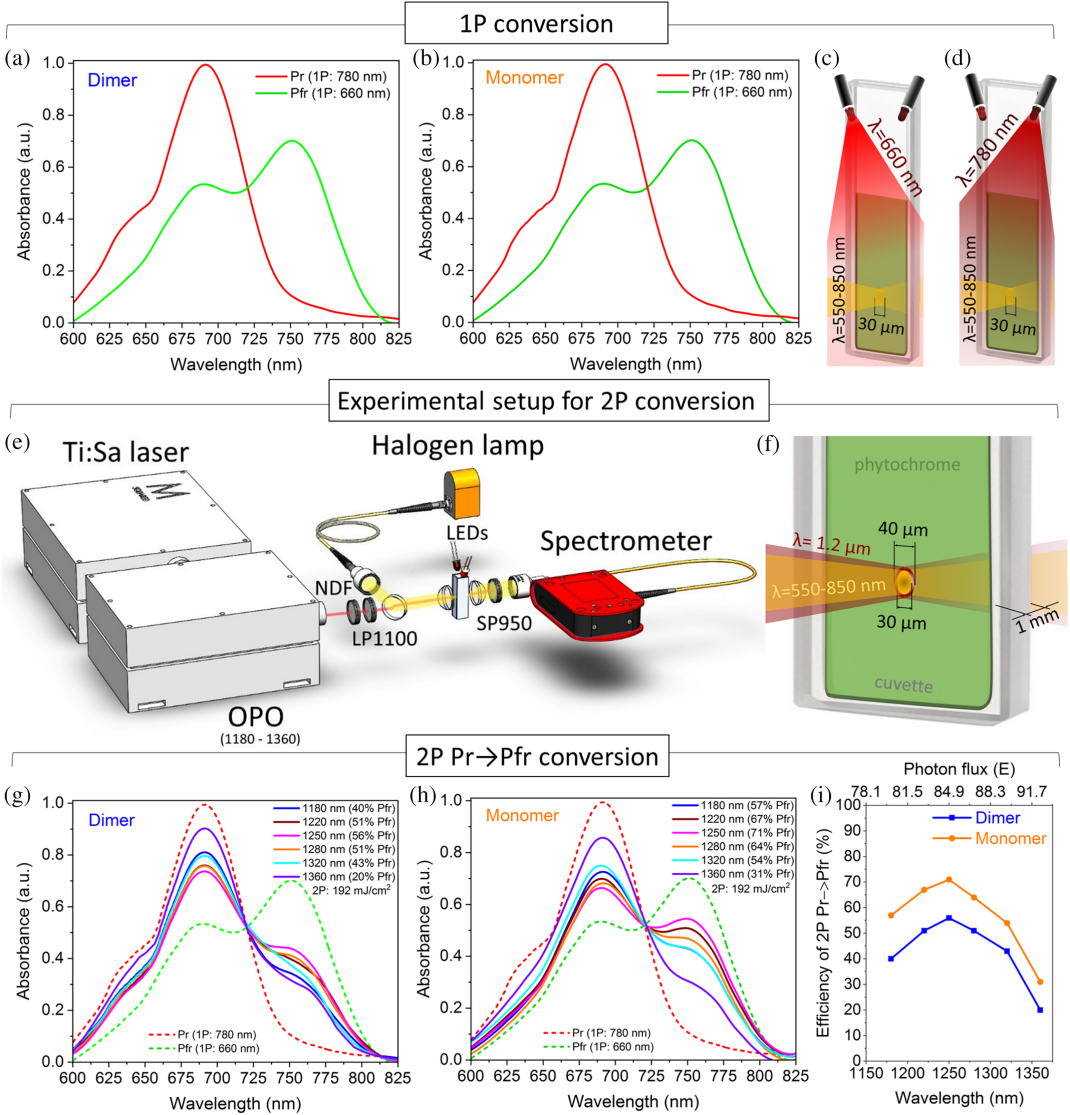
Single‐photon (1P) and two‐photon (2P) conversion. (a) Absorption spectra of dimer and (b) monomer after 1P conversion to the Pfr (green line) and Pr (red line) states upon illumination with (c) 660 nm and (d) 780 nm LEDs, respectively. (e) Experimental setup for 1P Pr → Pfr, Pfr → Pr and 2P Pr → Pfr photoconversion. (f) Schematic representation of a sample cuvette illuminated by co‐directed lamp and laser beams for spectral measurements of 2P Pr → Pfr photoconversion. (g) Absorption spectra of dimer and (h) monomer under 2P Pr → Pfr photoconversion with 1180–1360 nm laser irradiation. (i) Efficiency of 2P conversion depending on laser wavelength.

The *Dr*BphP‐PCM exhibits two absorbance states, named Pr and Pfr (Figure 1e). We studied the photoconversion between these states for monomer and dimer with the same concentration of 60 μM. Since samples could have unbound biliverdin molecules or different stages of denaturation, we investigated their linear optical properties: 1P Pr → Pfr and Pfr → Pr conversions (Figures 1e and 2a,b).

In 1P Pr → Pfr photoconversion experiments, *Dr*BphP‐PCM samples were illuminated by 660 nm light emitting diode (LED) (Figure 2c) (details in Section 4). This light illumination transferred both samples to the Pfr state, which has a prominent peak in the absorption spectra at 760 nm (Figure 2a,b).

The absorbance of the samples was calculated as $A=\log\left(I_{0}/I\right)$, where $I_{0}$ is the incident light from the halogen lamp passed through the empty cuvette, and I is the light passed through the sample and recorded by the spectrometer. The absorption spectra of the Pfr states for the dimer and monomer are shown as green lines in Figure 2a,b, which are in agreement with previously reported spectra (Lychagov et al., 2016; Takala et al., 2015).

The absorption spectra of Pr states (780 nm LED illumination) were repeated using the same technique with a halogen lamp and a spectrometer (Figure 2d,e). The spectra of dimer and monomer show a noticeable absorption peak at a wavelength of 690 nm (Figure 2a,b, red lines), which indicates a transition from the Pfr to the Pr state. Experiments with reversible photoconversion were repeated several times with different illumination times, and no signs of changes in the intensity of the absorption peaks in the spectra were noted.

Similar to the dimer behavior, the monomer has a reversible photoconversion, even in the absence of a protomer partner (Figure 1c). In the case of the dimer, there are large structural rearrangements with a twist of the PHY domain and increase/decrease of PHY‐PHY distance between protomers for Pr → Pfr and Pfr → Pr conversions, respectively (Burgie et al., 2016; Takala et al., 2014). The PCM structural rearrangements of monomer are similar: bend and twist of the PHY domain relative to the PAS‐GAF domains with slight differences in the Amide I and II regions (Takala et al., 2016). Both dimer and monomer have a light‐dependent transformation of the BV chromophore, where the ring D of BV undergoes a 180‐degree rotation, transitioning between two isomeric forms known as the *Z*‐ and *E*‐configurations of its C15=C16 double bond, corresponding to the Pr and Pfr states (Figure 1d).

#### 2.1.2 Nonlinear optical properties

The 1P Pr → Pfr conversion can be arranged by 630–680 nm illumination (energy of 1.88 eV), while nonlinear 2P conversion requires two photons of lower energy of around 0.94 eV (~1320 nm) to be absorbed.

To determine the optimal laser wavelength, we studied the 2P Pr → Pfr conversion efficiency of dimer and monomer depending on different excitation wavelengths (1180–1360 nm), maintaining a constant average power of 5.1 mW, which is equivalent to an energy fluence of 192 mJ/cm^2^ (Figure 2e,f) (details in Section 4). To ensure homogeneous experimental conditions, each evaluation of the absorption spectra was preceded by a 2‐min 1P Pfr → Pr conversion using a 780 nm LED. The sample temperature was monitored using a thermocouple, ensuring that it did not increase by more than 0.5°C near the laser beam spot. The absorption spectra of dimer and monomer samples after 2P Pr → Pfr conversions are shown in Figure 2g,h.

Both samples demonstrate gradual changes in 2P Pr → Pfr conversion efficiency, depending on the wavelength of absorbed light. All spectra show a decrease in the peak at 690 nm and a slight absorption increase at 720–775 nm (Figure 2g,h), which is unique for the Pfr state (Figure 2a,b, green lines). The experimentally measured absorption spectra exhibit slight variations in shape, which may be attributed to sample diffusion within the studied volume, as well as the effects of spectral averaging.

Since the changes in absorption spectra are complex, including the disappearance of the peak at 690 nm and rise at 625–650 nm, slight changes at 675 nm, and increase at 720–775 nm, the efficiency of the 2P conversion *η* from the Pr to the Pfr state was estimated by the standard method of spectral difference (SD) between sample absorbance after 2P and 1P conversion ${\mathrm{SD}}_{2P\;\mathrm{Pfr},\;1P\;\mathrm{Pfr}}$(Teska et al., 2013):

(1) $$\eta =\frac{\left({\mathrm{SD}}_{1P\;\mathrm{Pr},\;1P\;\mathrm{Pfr}}-{\mathrm{SD}}_{2P\;\mathrm{Pfr},\;1P\;\mathrm{Pfr}}\right)}{{\mathrm{SD}}_{1P\;\mathrm{Pr},\;1P\;\mathrm{Pfr}}}\times 100\%,$$

where $SD_{1P\;\mathrm{Pr},\;1P\;\mathrm{Pfr}}$ is the SD between the absorbance of Pr and Pfr states after sample illumination by 780 and 660 nm LEDs.

SD can be found by the formula shown in Equation (2):

(2) $$\mathrm{SD}=\sqrt{\frac{\sum_{\lambda_{i}=\lambda_{\min}}^{\lambda_{\max}}{\left(A_{1\lambda_{i}}-A_{2\lambda_{i}}\right)}^{2}}{n}},$$

where $A_{1\lambda_{i}}$ and $A_{2\lambda_{i}}$ are absorbance values of comparing spectra in the wavelength of $\lambda_{i}$. The $\lambda_{i}$ can be varied in the wavelength range of *λ*_min_–*λ*_max_ with several data points n in the range of interest.

Under 1360 nm laser illumination, the dimer exhibits a 20% efficiency of 2P Pr → Pfr conversion (Figure 2g, violet line) compared to the Pfr absorption spectra of phytochrome after 1P Pr → Pfr (Figure 2a, green line) that was counted as a reference efficiency. The nonlinear conversion efficiency of this sample increases to 51% after illumination by 1220 and 1280 nm light (Figure 2g, wine, orange lines) and reaches a maximum of 56% after laser irradiation by 1250 nm (Figure 2g, navy blue line).

However, the monomer demonstrates higher efficiency with 2P Pr → Pfr conversion, which is noticeable by the appearance of an absorption peak at 760 nm (Figure 2h). The excitation wavelengths from 1180 to 1280 nm demonstrate a better efficiency of 2P Pr → Pfr conversion (>57%) compared to the highest efficiency of the dimer sample (56% at 1250 nm; Figure 2g). Pulsed laser irradiation with a wavelength of 1250 nm and average power of 5.1 mW provides a 2P Pr → Pfr conversion efficiency of 71% (Figure 2h, pink line).

Although 1P illumination at 660 nm effectively converts the Pr to Pfr state, 2P irradiation at the twice longer wavelength, 1320 nm (Figure 2g,h, aqua blue lines), is less effective than 1220–1280 nm wavelengths (Figure 2g,h, wine, pink, orange lines). This behavior can be explained by elevating the reverse process of Pr → Pfr conversion (Sokolovski et al., 2021). Therefore, the optimum wavelength range of 2P conversion is slightly shifted and reaches maximum efficiency at 1250 nm (Figure 2i), which is 56% for the dimer and 71% for the monomer.

**FIGURE 3:**
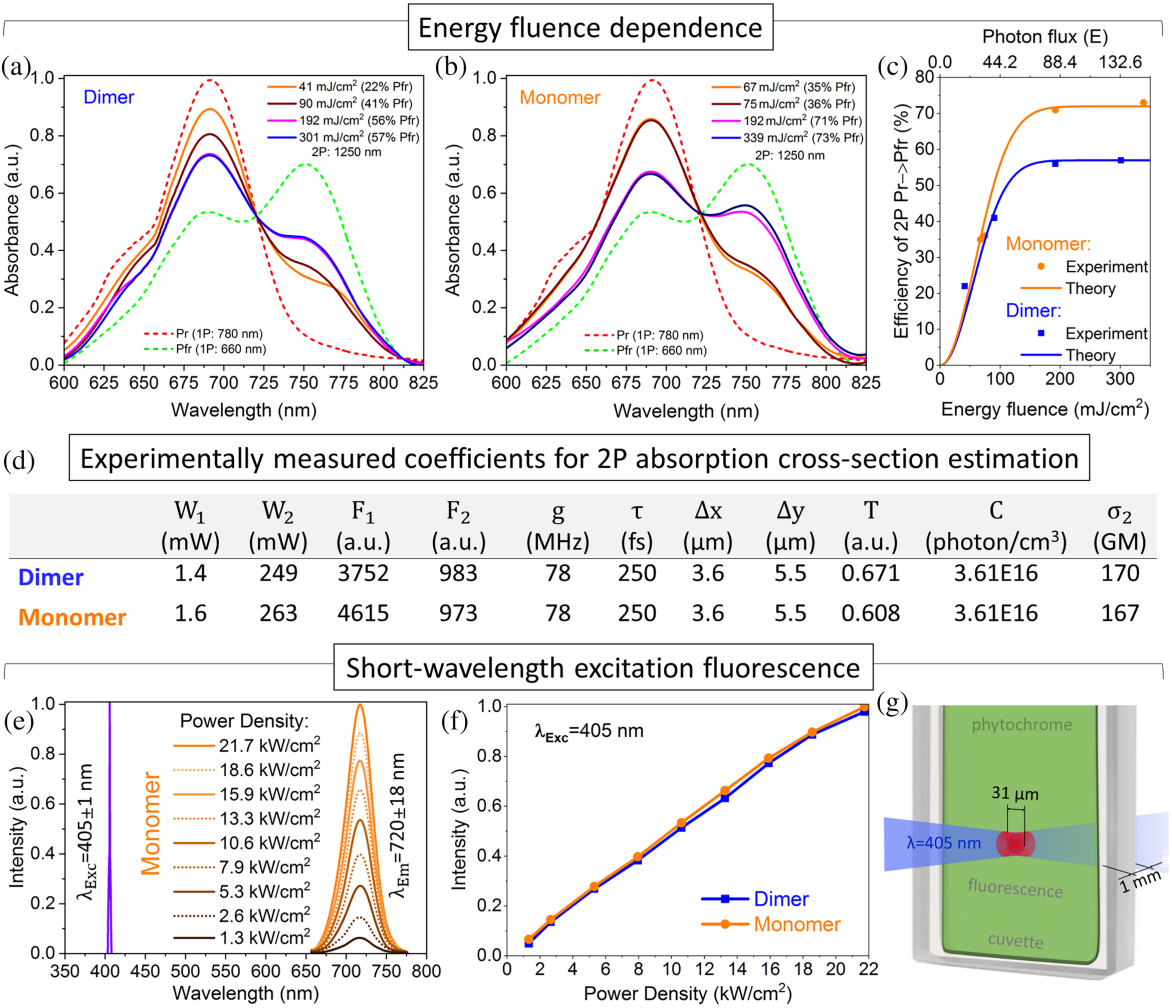
Two‐photon (2P) Pr → Pfr conversion depending on energy fluence and short‐wavelength excitation (SWE) fluorescence. (a) Absorption spectra of dimer and (b) monomer depending on laser energy fluence (1250 nm). (c) Experimental and theoretical results of 2P conversion efficiency under 1250 nm irradiation, depending on laser energy fluence. (d) Experimentally measured values of fluorescence emission intensity and laser beam parameters required for the estimation of the 2P absorption cross‐section. (e) 405 nm continuous wave (CW) laser used as SWE laser source and fluorescence emission spectra of monomer depending on the laser power density. The emission spectra were recorded after passing through a 725 ± 50 nm band‐pass filter. (f) SWE fluorescence emission intensity of monomer and dimer depending on the power density (1.3–21.7 kW/cm^2^). (g) Schematic representation of a sample cuvette illuminated by 405 nm CW laser for spectral measurements of SWE fluorescence.

The efficiency can be further improved by increasing the laser energy fluence (Figure 3a,b). For example, the laser energy fluency of 339 mJ/cm^2^ (average power of 9 mW) increases the Pfr yield and the efficiency of 2P Pr → Pfr conversion of monomer to 73% (Figure 3b).

In the case of the dimer, it exhibits a lower rate of conversion to the Pfr form under 2P absorption (2PA) compared to the monomer at equivalent concentrations. The pulsed laser illumination at a wavelength of 1250 nm with an energy fluence range of 41–301 mJ/cm^2^ leads to an increase in the 2P Pr → Pfr conversion efficiency from 22% to only 57% (Figure 3a,c). The experimental results were confirmed by theoretical modeling of the 2P conversion of dimer and monomer.

#### 2.1.3 Computational analysis

In our previous work, we used a quantitative analysis of 2P conversion and demonstrated that in addition to the 2P Pr → Pfr reaction, there is a competitive reverse Pfr → Pr process, asymptomatically leading to a plateau for the Pfr yield (Sokolovski et al., 2021).

Here, instead of empirical estimation of photoconversion rate constants, we applied a molecular mechanical analysis based on the experimentally measured photon flux and 2PA cross‐section.

Photon flux *φ* is defined as the number of photons per second and unit area on a surface and given in Einstein units ($\frac{photons}{s\times m^{2}}$). Photon flux inside the focal volume directly depends on the applied level of laser intensity *I* and wavelength *λ*:

(3) $$\varphi =\frac{\mathrm{I\lambda}}{{\mathrm{hcN}}_{A}}=\frac{2\mathrm{P\lambda}}{\pi r^{2}\mathrm{hc}N_{A}},$$

where *h*—Planck constant, *N*_A_—Avogadro constant, and *c*—speed of light. In the experiments, the laser wavelength *λ* was 1250 nm, beam radius *r* was 20 μm, while the power level *P* was varied from 1.1 mW to 9.0 mW, resulting in the photon flux values of 18.31–149.79 E.

The value of 2PA cross‐section *δ* was found by measuring 1P and 2P fluorescence emission intensity (*F*_1_ and *F*_2_, respectively), represented in Equation (4) (Makarov et al., 2008).

(4) $$\delta =\frac{1}{\sqrt{2}}{\left(\frac{\pi }{\ln2}\right)}^{3/2}\frac{W_{1}}{W_{2}^{2}}\frac{\mathrm{hc}\lambda_{1}}{{\lambda_{2}}^{2}}\frac{F_{2}}{F_{1}}\mathrm{g\tau}\Delta x\Delta y\frac{\left(1-T\right)}{\mathrm{lC}},$$

In Equation (4), *W*_1_, *W*_2_ and *λ*_1_, *λ*_2_ are lasers' average power and operating wavelengths for 1P and 2P fluorescence excitation, respectively. Parameters of 2P excitation are included in a pulse repetition rate *g* and a temporal pulse width τ, while spatial beam widths and beam pathway are *Δ* *x*, *Δ* *y*, and l. The left coefficients, *T* and *C* are sample transmittance and concentration, respectively.

During the experiments, the 1P and 2P fluorescence emission spectra were obtained by using a ($\lambda_{1}=405\;\mathrm{nm}$) continuous wave (CW) laser and ($\lambda_{2}=810\;\mathrm{nm}$) Ti:Sapphire laser focused on the same spot in a 1 mm (l) thick sample cuvette. The profile and beam size were controlled by a laser beam profile camera.

The dimer sample has a bigger value of 2PA cross‐section (*δ* = 170 GM) than the monomer (*δ* = 167 GM). The values of coefficients using in Equation (4) are presented in Figure 3d.

Knowing photon flux, 2PA cross‐section, and reverse back coefficient *k*_rb_ (empirically founded from Sokolovski et al. 2021), one can find the number of molecules excited per unit of time and volume $n_{m}^{\left(2\right)}$ (Equation 5) (Rausenberger et al., 2010):

(5) $$n_{m}^{\left(2\right)}=\frac{1}{2}\;k_{\mathrm{rb}}\delta N_{g}\phi^{2}.$$

At each time *t*, $n_{m}^{\left(2\right)}$ has to be the opposite of the change in the population density for stage *g* between time *t* and *t* + *dt*, because the total number of molecules is conserved:

(6) $$n_{m}^{\left(2\right)}=-\frac{{\mathrm{dN}}_{g}}{\mathrm{dt}}.$$

Thus, Equation (5) becomes

(7) $$\frac{{\mathrm{dN}}_{g}}{\mathrm{dt}}=-\frac{1}{2}\;k_{\mathrm{rb}}\delta N_{g}\phi^{2},$$

which, after integration between *t* = 0 and *t = τ* and using $N_{g}\left(0\right)=N_{0}$, gives Equation (8):

(8) $$N_{g}\left(\tau \right)=N_{0}e^{-1/2\;k_{\mathrm{rb}}\delta \phi^{2}\tau }.$$

The total number of molecules excited (per unit volume) during each pulse is thus:

(9) $$\begin{array}{rcl} N_{m}^{\left(2\right)}\left(\tau \right) & = & N_{m,2\mathrm{PA}}^{\left(2\right)}\left(\tau \right)+N_{m,2\mathrm{PF}}^{\left(2\right)}\left(\tau \right)\\ & = & N_{0,2\mathrm{PA}}+N_{0,2\mathrm{PF}}-\left(N_{g,2\mathrm{PA}}\left(\tau \right)+N_{g,2\mathrm{PF}}\left(\tau \right)\right)\\ & = & \left(N_{0,2\mathrm{PA}}+N_{0,2\mathrm{PF}}\right)\left(1-e^{-\left(1/2\right)k_{\mathrm{rb}}\delta \phi^{2}\tau }\right). \end{array}$$

It is worthwhile noting, that Equation (9) accounts for both mechanisms under consideration (Piatkevich et al., 2013; Rausenberger et al., 2010).

Equation (5) can also be viewed as a definition of the 2PA cross‐section. The factor of 1/2 in Equation (5) accounts for the fact that two absorbed photons produce one excited molecule. The fraction of molecules excited per pulse can be derived from Equation (9) as

(10) $$\Delta_{m}^{\left(2\right)}=\frac{N_{m}^{\left(2\right)}\left(\tau \right)}{N_{0}}=1-e^{-\left(1/2\right)\;k_{\mathrm{rb}}\delta \phi^{2}\tau },$$

which, if the exponent of *e* is small, becomes

(11) $$\Delta_{m}^{\left(2\right)}\approx \frac{1}{2}\;k_{\mathrm{rb}}\delta \phi^{2}\tau .$$

Therefore, the approximation corresponds to a scenario in which only a small fraction of molecules is excited per pulse. Consequently, the ground state of the system remains largely populated and is not significantly depleted during the pulse. It is worthwhile noting that saturation is observed because all the molecules have excited during the pulse of the duration from *t* = 0 till *t* = *τ*. It also should be noted that saturation level is strongly affected by the 2P fluorescence effect (Figure 3c).

Molecular mechanics uses classical mechanics to model molecular systems. The molecular mechanical formulation presented in this work is in good agreement with the experimental results demonstrating a plateau dynamic of the Pfr yield. In other words, 2P Pr → Pfr conversion is limited to a certain level (57% for dimer and 73% for monomer) which cannot be increased by applying a higher laser power or longer illumination time due to the elevated Pfr → Pr reversion.

The molecular mechanics formulation is a vast simplification and only an approximation. One would need to verify through quantum calculations on small systems that molecular mechanics parameters describe that process in a satisfactory manner. At that point, one may have some confidence that whatever has been inferred from the large calculations has some basis in fact. To conclude, it is worthwhile mentioning that the model based on the quantum mechanics approach describes the system in a more precise way (Sokolovski et al., 2021).

### 2.2 Fluorescence

#### 2.2.1 Short‐wavelength excitation

As previously discussed, bacterial phytochromes exhibit an absorption peak near 690 nm, corresponding to the Q‐band of their absorption spectrum (Figure 1e). Excitation with a 640–700 nm laser can induce fluorescence emission with a central wavelength typically within a range of 670–720 nm. Moreover, fluorescence properties can also be induced by the absorption of light in the Soret band, which falls within the range of 390 ± 30 nm (Rumyantsev et al., 2015).

**FIGURE 4:**
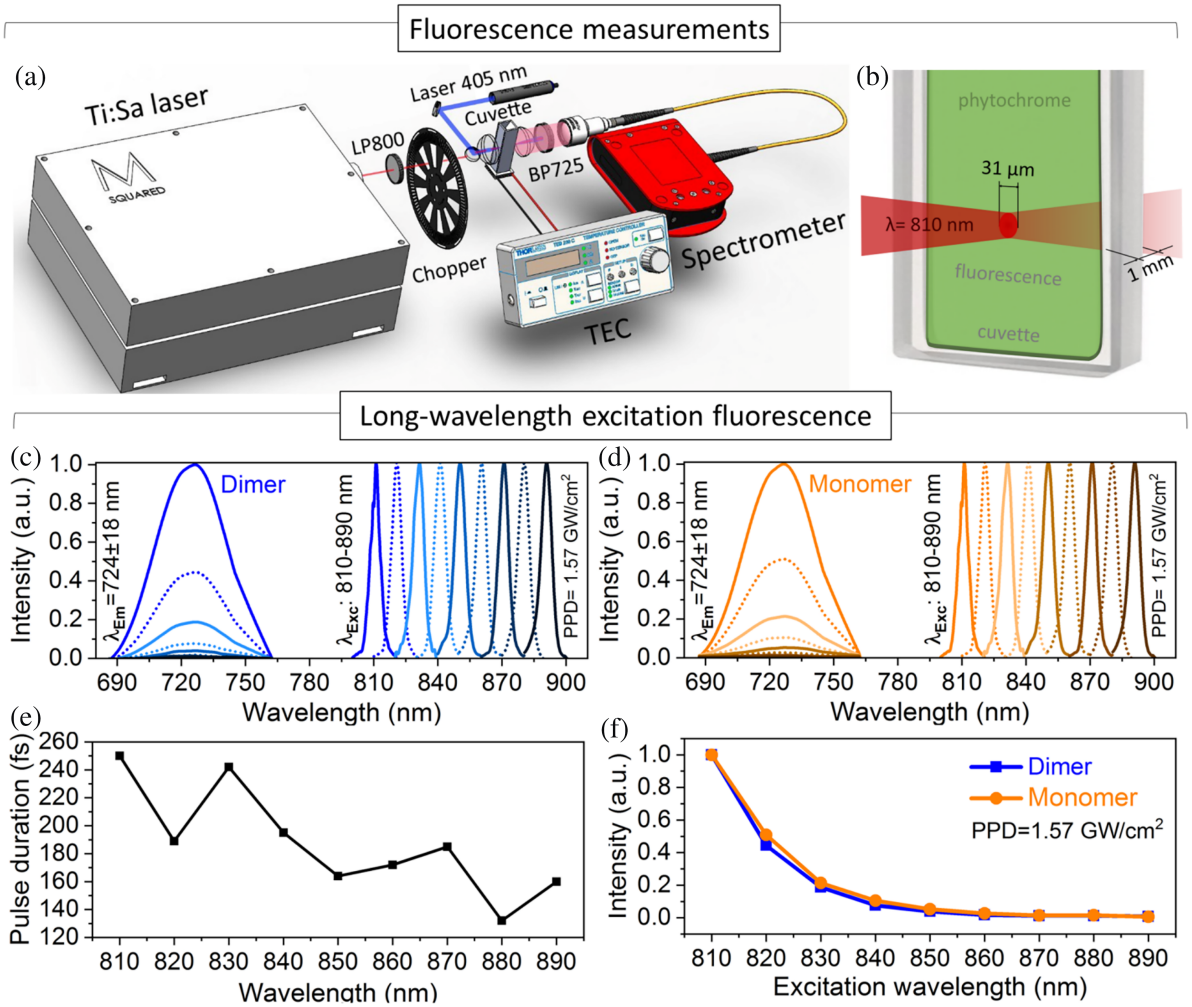
Long‐wavelength excitation (LWE) fluorescence. (a) Experimental setup for short‐wavelength excitation (SWE) and LWE fluorescence measurements. (b) Schematic representation of a sample cuvette illuminated by 810 nm pulsed laser for spectral measurements of LWE fluorescence emission. (c) LWE fluorescence emission intensity of dimer and (d) monomer under 810–890 nm light with a peak power density (PPD) of 1.57 GW/cm^2^. (e) Pulse duration of Ti:Sapphire laser depending on laser wavelength. (f) LWE fluorescence emission intensity depending on excitation wavelength with a constant PPD of 1.57 GW/cm^2^.

To measure the SWE fluorescence (Figure 3e,f), a 405 nm CW laser focused to a 31 μm diameter beam spot onto a 1 mm sample cuvette (Figure 3g). The CW laser used in these experiments had an average power of 5–82 mW, resulting in a power density of 1.3–21.7 kW/cm^2^. To separate the excitation and emission signals, a 725 ± 50 nm band‐pass (BP) filter was placed behind the cuvette (Figure 4a). The sample fluorescence emission was then collected and recorded using a spectrometer (details in Section 4).

Upon SWE, dimer and monomer exhibit fluorescence emission, featuring a distinct emission peak at a NIR‐I wavelength region of 720 ± 18 nm (Figure 3e). The SWE fluorescence intensity of the monomer and dimer linearly grows with the increase of the incident laser power density and remains comparable under identical experimental conditions (Figure 3f). These results are in agreement with well‐studied 1P fluorescent properties of dimer and monomer *Dr*BphP‐PCM (Lehtivuori et al., 2013; Lychagov et al., 2016; Shcherbakova et al., 2016).

#### 2.2.2 Long‐wavelength excitation

The study of NIR‐I LWE fluorescence of dimer and monomer started from a laser wavelength of 890 nm with a systematic decrease to 810 nm (Figure 4a,b). For each measurement, the sample was exposed to femtosecond pulsed laser radiation for a time not exceeding 2 min, during which fluorescence emission was recorded. The integration duration of 15 s was set for each recording.

The laser illumination at these wavelengths induces fluorescence emission at 724 ± 18 nm wavelength (Figure 4c,d). All fluorescence emission spectra were normalized to the maximum fluorescence intensity observed at the excitation wavelength of 810 nm. Throughout the measurements, a consistent peak power density was maintained across different wavelengths (810–890 nm) using neutral density filters, compensating for variations in laser pulse duration (Figure 4e).

Figure 4f demonstrates the dependence of the fluorescence emission of dimer (blue line) and monomer (orange line) on the excitation wavelength spanning 810–890 nm with a constant peak power density of 1.57 GW/cm^2^. As the excitation wavelength gradually decreases from 890 to 810 nm, there is an increase in fluorescence brightness. This phenomenon can be attributed to the reduced light absorption at the longer near‐infrared wavelengths.

**FIGURE 5:**
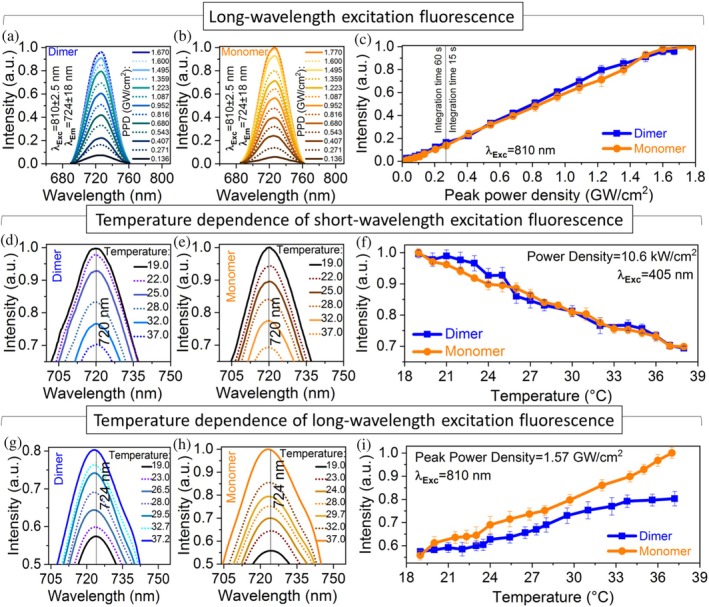
Dependence of long‐wavelength excitation (LWE) fluorescence on peak power density and temperature dependence of short‐wavelength excitation (SWE) and LWE fluorescence emission intensities. (a) LWE fluorescence emission spectra (*λ*_Exc_ = 810 nm, *λ*_Em_ = 724 nm) of dimer and (b) monomer depending on peak power density. (c) LWE fluorescence emission intensity depending on peak power density (27 MW/cm^2^ to 1.77 GW/cm^2^). (d) Zoomed SWE fluorescence emission spectra (*λ*_Exc_ = 405 nm, *λ*_Em_ = 720 nm) of dimer and (e) monomer depending on the sample temperate controlled by an external heater. (f) SWE fluorescence emission intensity depending on temperature (19–38°C). (g) Zoomed LWE fluorescence emission spectra (*λ*_Exc_ = 810 nm, *λ*_Em_ = 724 nm) of dimer and (h) monomer depending on the sample temperate controlled by an external heater. (i) LWE fluorescence emission intensity depending on temperature (19–38°C).

To understand the nature of LWE fluorescence, the emission intensity was studied as a function of the laser peak power (Figure 5a,b). The range of peak power density was adjusted from 27 MW/cm^2^ to 1.77 GW/cm^2^ (2–130 mW for 810 nm excitation wavelength). Fluorescence intensity measurements under light excitation with a peak power density greater than 0.27 GW/cm^2^ were recorded using a spectrometer with an integration time of 15 s. Conversely, experiments with peak power densities below this value were measured with an integration time of 60 s, increasing a laser excitation period to 4 min per curve point.

Figure 5a,b illustrates the relationship between LWE fluorescence emission and the peak power density of the excitation laser operating at a wavelength of 810 nm with a pulse duration of 250 fs. In both samples, the increase of peak power density leads to a linear rise in fluorescence brightness until a value of 1.5 GW/cm^2^. Beyond this threshold, the fluorescence intensity gradually changes the trend, reaching a plateau (Figure 5c). The complex power dependence may result from multiple processes contributing to fluorescence emission. Since the power dependence is not quadratic, it is unlikely that the mechanism is classical two‐photon excitation even though the excitation wavelengths (810–890 nm) are double the Soret band absorption (390 ± 30 nm) and red‐shifted by 120–200 and 90–170 nm from the absorption maximum of the Pr state (690 nm) and the fluorescence emission peak (724 nm), respectively (Gryczynski et al., 2002). The linear power dependence can be attributed to entangled two‐photon absorption or a mix of 1P and 2P absorption processes, while the emission intensity plateau can reflect on the saturation of the excited level and the combination of several mechanisms in energy dissipation (Drobizhev et al., 2003; Gryczynski et al., 2002; Schlawin et al., 2018).

#### 2.2.3 Temperature dependence of fluorescence intensity

SWE and LWE processes exhibit distinct mechanisms of light emission that can also be noticed through experiments assessing their temperature‐dependent fluorescence emission intensity.

To measure the fluorescent emission property as a function of temperature, the room temperature was kept constant at 22°C. The Peltier thermal controller positioned beneath the sample cuvette was used to gently warm the sample from 19 to 38°C, with the upper limit aligning with the average temperature of the human brain (Rzechorzek et al., 2022).

A 405 nm CW laser with an average power output of 40 mW (a power density of 10.6 kW/cm^2^), Peltier element, and thermocouple were used to measure temperature dependence of SWE fluorescence emission intensity. Each spectral measurement of the samples under 1P laser excitation was completed within 1 min to prevent any additional heating caused by the laser irradiation. During the experiment, as the temperature of the samples gradually increased by the Peltier element, both monomer and dimer displayed a linear reduction in their fluorescence intensity (Figure 5f). At a temperature of 38°C, the fluorescence intensity dropped to one‐third of the initial spectral measurements taken at a sample temperature of 19°C. The results align with experimental data on the temperature dependence of 1P fluorescence emission intensity for various phytochromes (Gryczynski et al., 2002; Njimona et al., 2014).

Remarkably, repetition of the experiment following the cooling of the samples, the fluorescence intensity rebounded to its initial level, underscoring the reproducibility of the experimental outcomes.

For measurements of LWE fluorescence emission depending on temperature, the experimental setup was replicated using the 810 nm Ti:Sapphire laser with a peak power density of 1.57 GW/cm^2^ (average power of 118 mW).

A gradual increase in sample temperature by an external heat source produced an opposite effect in the LWE fluorescent intensity (Figure 5i). In the 19–23°C temperature range, both samples showed a slight increase in fluorescence emission intensity. The temperature elevation from 23 to 37°C leads to a 25% increase in fluorescence emission intensity under LWE excitation for the dimer (Figure 5i, blue line). The monomer exhibited even higher fluorescence intensity, with nearly a twofold increase in emission brightness at 37°C compared to the measurements at 19°C (Figure 5i, orange line).

Despite the similar fluorescence emission intensity level of monomer (0.56 a.u.) and dimer (0.58 a.u.) samples under 19°C, the monomer demonstrates a more significant temperature dependence of light emission. The fluorescence intensity of the monomer form increased by 39% more than the traditional dimer form at a temperature of 37°C. It is noteworthy that further gradual cooling to room temperature and repeating the experiments show similar correlated data.

The different temperature dependencies observed in the fluorescence emission under 405 and 810 nm wavelength excitation heated by external source suggest the dominance of different underlying mechanisms (Gryczynski et al., 2002). Although these mechanisms require additional study, it has been hypothesized in the literature that fluorescence quantum yield and the quantum efficiency of chromophore isomerization due to photo‐ and thermos‐conversion are intricately interrelated (Ihalainen et al., 2015; Nagano et al., 2022). In the case of SWE and heating, we assume that conformational changes serve as the primary pathway for energy dissipation. The increased temperature may cause partial unfolding of the PHY domain, disrupting the chromophore hydrogen bonding network and leading to its deprotonation (Njimona et al., 2014). In contrast, under LWE, external heating adds additional energy to the system, increasing the number of excited molecules and, therefore, enhancing fluorescence emission intensity. This unusual temperature dependence of fluorescence emission under LWE was noticed in several studies of 1P and 2P excitation fluorescence (Gryczynski et al., 2002; Mikhaylov et al., 2022; Zhang et al., 2021). One possible explanation is an additional hot‐band absorption effect, which can occur if the excitation source has a broad operating spectrum, causing the absorbed energy to populate vibrational levels of the degenerate ground state (*S*_0_) (Mikhaylov et al., 2022; Zhang et al., 2021). Moreover, it is possible that after fluorescence emission, the system relaxes back to a non‐zero vibrational level of the ground state (*S*_0_ *υ*_1_, *υ*_2_, …). This process could explain the observed difference in emission wavelengths between SWE and LWE fluorescence (Figure 5d,e,g,h). However, this hypothesis requires further investigation.

## 3 DISCUSSION

The experimental findings and theoretical model illustrate the linear and nonlinear optical properties of the truncated *Dr*BphP dimer and monomer variants. The monomer demonstrates greater efficiency of the 2P Pr → Pfr conversion (73%) compared to the dimer (57%).

When the absorbed energy of two photons is low (less than 40 mJ/cm^2^), the dimer PCM configuration has an advantage in the mutual support of protomers due to the energy homotransfer between the protomers in the dimer during transitions between the Pr and Pfr states. The gradual rotation of the BV ring D in one protomer catalyzes the BV transition in the second protomer toward its *E*‐configuration. The joint distribution of the absorbed energy between these protomers initiates a chain reaction between the two BV chromophores, gradually increasing the Pfr yield in the dimer.

In contrast, for the monomer sample, this level of energy fluence is not enough for the 2P conversion. Only when the energy fluence threshold for an independent monomer reaches 60 mJ/cm^2^, the monomer facilitates the immediate process of the D‐ring rotation and demonstrates a higher 2P Pr → Pfr conversion compared to the dimer (Figure 3c).

As the energy fluence continues to increase, the difference in the Pfr yields between dimer and monomer becomes more noticeable. For instance, under 1250 nm illumination with an energy fluence of 192 mJ/cm^2^, the dimer and monomer samples reach the Pfr yields of 56% and 71%, respectively (Figure 3c). This apparent difference in 2P conversion efficiency can be attributed to the fact that in the dimer, the absorbed energy aside from contributing to D ring rotation and PHY domain twist, involves the separation of PHY domains of protomers, expanding the size of the molecule from 85 to 90 Å (Burgie & Vierstra, 2014).

The monomer achieves its highest Pfr yield and efficiency in the 2P conversion of 73% when exposed to 1250 nm (NIR‐II) laser light with an energy fluence of 339 mJ/cm^2^. A further increase in laser power or prolongation of the illumination time was not accomplished with a rise in efficiency, which was confirmed by the computational analysis.

This work demonstrates, for the first time, 2P photoconversion and SWE and LWE fluorescence emission of a monomeric bacterial phytochrome, such as *Dr*BphP‐PCM. The superior performance of monomeric *Dr*BphP‐PCM, with substantially higher 2P photoconversion efficiency and increased LWE fluorescence brightness at physiological temperatures compared to its dimeric counterpart, positions it as an ideal template for designing NIR monomeric fluorescent proteins and optogenetic tools. NIR‐II nonlinear conversion has the potential to significantly enhance precise optogenetic stimulation, while NIR‐I fluorescence excitation and emission can enable deeper tissue penetration, improving the spatial resolution of imaging (Dremin et al., 2022; Galiakhmetova et al., 2023; Ilic et al., 2006).

Looking forward, this work lays the foundation for several promising translational applications. In the field of optogenetics, a monomer potentially can be used as a long‐wavelength activated Forster resonance energy transfer donor based on so‐called bathy phytochromes, such as based on RpBphP1, in which the Pfr state is thermodynamically stable (Kaberniuk et al., 2016; Redchuk et al., 2017). In biomedical imaging, the PCM's enhanced properties could lead to improved photoacoustic computed tomography probes for deep‐seated tumors and organs. Furthermore, the insights gained from this study could contribute to the development of novel NIR biosensors and light‐controlled enzymes, expanding the toolkit for synthetic biology and metabolic engineering.

## 4 MATERIALS AND METHODS

### 4.1 Clonning, expression and purification of proteins

The genes of monomer and dimer *Dr*BphP were provided by J. Ihalainen (University of Jyväskylä, Finland). For bacterial expression, the PCM encoding part of the *Dr*BphP genes was PCR‐amplified as a *Bgl*II–*Hind*III fragment and cloned into a pBAD/HisB vector (Life Technologies/Invitrogen). A LMG194 *Escherichia coli* bacterial host (Life Technologies/Invitrogen) was used for protein expression. A pWA23h plasmid encoding heme oxygenase (HO) from *Bradyrhizobium ORS278* (hmuO) under the rhamnose promoter was co‐transformed with a pBAD‐HisB plasmid encoding monomer or dimer *Dr*BphP‐PCM with a polyhistidine tag. The bacterial cells were grown in a restricted medium (RM) supplemented with ampicillin, kanamycin, and 0.02% rhamnose at 37°C for 6–8 h, followed by induction of protein expression by adding 0.002% arabinose and incubation for 24 h at 18°C. The protein was purified with Ni‐NTA agarose (Qiagen). The sample was desalted using PD‐10 columns (GE Healthcare). For optical measurements, the *Dr*BphP‐PCM monomer and dimer samples were concentrated to 60 μм and frozen at −80°C. Protein concentration was estimated based on the extinction coefficient of the Q‐band of the purified monomeric and dimeric *Dr*BphP‐PCMs. In turn, the extinction coefficients were calculated from a comparison of absorbance values at the Q‐band with the absorbance value at the Soret band at around 390 nm, assuming the latter had an extinction coefficient of free biliverdin of 39,900 $M^{-1}{\mathrm{cm}}^{-1}$. Before experiments, samples were thawed to room temperature. Each sample was transferred into a quartz cuvette with a thickness of 1 mm. Over the 2 h of the experiment, the sample conditions were controlled by measuring absorption spectra.

### 4.2 Optical experimental setup

The experimental studies of the 1P Pr → Pfr and Pfr → Pr conversions were conducted using 660 ± 7 nm and 780 ± 14 nm LEDs with an output power of 15 mW (LED660L, LED780L, Thorlabs). To ensure complete conversion of the sample, the LED illuminated continuously toward a cuvette containing the phytochrome sample (Figure 2c,d) for 2 min under complete dark conditions.

The absorption spectra were measured using: (1) Fourier transform infrared (FT‐IR) spectrometer (Vertex 80, Bruker); (2) spectrometer (HR4000CG‐UV–NIR, Ocean Optics) and a halogen lamp as the light source, directed onto the quartz cuvette and focused into a beam spot with a diameter of 30 μm.

To study the 2P Pr → Pfr conversion, a 50‐fs 4225‐Hz Ti:Sapphire laser (Astrella, Coherent) in conjunction with an optical parametric amplifier (TOPAS‐Prime, Coherent) was used. The laser operating wavelength could be tuned in the range of 1180–1360 nm. The laser beam was aligned with the halogen lamp beam, resulting in a focused spot size with a 40 μm diameter.

The experimental procedure involved two steps conducted in a dark environment. (1) 1P Pfr → Pr conversion was induced using LED illumination with a 780 nm wavelength for 2 min. (2) 2P Pr → Pfr conversion was initiated by using a NIR‐II pulsed laser for 30 s. To prevent photoconversion effects caused by halogen lamp illumination, the lamp beam was blocked during LED and NIR‐II laser irradiation and unblocked only for spectral measurements. The potential influence of the halogen lamp on photoconversion can be excluded, as spectral measurements showed no changes in the sample spectra after 30 s of illumination with the halogen lamp alone.

The transmitted light was subsequently filtered through a 900 nm short‐pass filter and collected by a spectrometer (Figure 2e,f). The efficiency of the 2P Pr → Pfr conversion was assessed by manually varying the laser wavelength in the range of 1180–1360 nm and adjusting the average power (1.1–9 mW) (energy fluency of 41–339 mJ/cm^2^).

For SWE and LWE fluorescence, the experimental setup included a 405 ± 1 nm CW laser (L405A1, Thorlabs) with an average power of 82 mW (a power density of 21.7 kW/cm^2^) and a co‐directed 810–890 nm 78 MHz Ti:Sapphire laser (Sprite XT, M Squared) (Figure 4a) with a pulse duration of 132–250 fs (Figure 4e). The average power was manually attenuated between 130 and 2 mW (peak power density of 1.77 GW/cm^2^ to 27 MW/cm^2^) measured after a 1 kHz frequency chopper. Both continuous and pulsed lasers were precisely aligned and focused onto a cuvette, with a spot diameter of 31 μm (Figures 3g and 4b).

Throughout the experiments, the cuvette remained open to facilitate the diffusion and was positioned on a Peltier element with a temperature regulation using Thorlabs thermoelectric cooler (TEC) Controller. The temperature of the phytochrome was continuously monitored via a thermocouple (TC‐08 data logger, Pico Technology). The room temperature remained constant at 22°C.

The fluorescence emission was separated from the excitation signal by a 725 ± 50 nm BP filter, collected through a collimator, and recorded using a high‐resolution spectrometer (HR4000CG‐UV–NIR, Ocean Optics). For SWE, a spectrometer integration time was set at a value of 100 ms, while for LWE, integration times of 15 and 60 s were used for a peak power density of 0.27–1.77 GW/cm^2^ and 0.027–0.27 GW/cm^2^, respectively.

## AUTHOR CONTRIBUTIONS

**Diana Galiakhmetova:** Investigation; methodology; visualization; conceptualization; writing – original draft; writing – review and editing; data curation; formal analysis; validation; resources. **Aleksandr Koviarov:** Conceptualization; methodology; investigation; validation; data curation; visualization; writing – review and editing; resources. **Viktor Dremin:** Conceptualization; methodology; investigation; validation; data curation; formal analysis; writing – review and editing; resources. **Tatjana Gric:** Writing – review and editing; conceptualization; methodology; software; data curation; resources; validation; investigation; formal analysis. **Dmitrii Stoliarov:** Writing – review and editing; conceptualization; methodology; investigation; data curation; formal analysis; validation. **Andrei Gorodetsky:** Investigation; validation; data curation; formal analysis; writing – review and editing; resources. **Marios Maimaris:** Writing – review and editing; investigation; validation; formal analysis; data curation; resources. **Daria M. Shcherbakova:** Writing – review and editing; methodology; investigation; data curation; conceptualization; validation; resources. **Mikhail Baloban:** Methodology; validation; writing – review and editing; formal analysis; data curation; investigation; conceptualization; resources. **Vladislav V. Verkhusha:** Supervision; writing – review and editing; writing – original draft; conceptualization; methodology; investigation; funding acquisition; resources. **Sergei G. Sokolovski:** Funding acquisition; methodology; investigation; conceptualization; supervision; project administration; writing – review and editing; resources. **Edik Rafailov:** Project administration; supervision; writing – review and editing; resources; funding acquisition.

## CONFLICT OF INTEREST STATEMENT

The authors declare no competing interests.

## Data Availability

The data that support the findings of this study are available from the corresponding author upon reasonable request.
